# Taxonomic study of the genus *Ischnothyreus* Simon, 1893 from Myanmar (Araneae, Oonopidae)

**DOI:** 10.3897/zookeys.993.57676

**Published:** 2020-11-16

**Authors:** Yanfeng Tong, Shuqiang Li, Dongju Bian

**Affiliations:** 1 Life Science College, Shenyang Normal University, Shenyang 110034, China Shenyang Normal University Shenyang China; 2 Southeast Asia Biological Diversity Research Institute, Chinese Academy of Sciences, Yezin, Nay Pyi Taw 05282, Myanmar Southeast Asia Biological Diversity Research Institute, Chinese Academy of Sciences Yezin Myanmar; 3 Institute of Zoology, Chinese Academy of Sciences, Beijing 100101, China Institute of Zoology, Chinese Academy of Sciences Beijing China; 4 CAS Key Laboratory of Forest Ecology and Management, Institute of Applied Ecology, Shenyang 110016, China Institute of Applied Ecology Shenyang China

**Keywords:** Goblin spider, morphology, new species, taxonomy

## Abstract

Seven new species of the genus *Ischnothyreus* Simon, 1893 from the spider family Oonopidae Simon, 1890 are reported from Myanmar: *I. hponkanrazi***sp. nov.** (♀), *I. jianglangi***sp. nov.** (♀), *I. meukyawwa***sp. nov.** (♂♀), *I. putao***sp. nov.** (♀), *I. qiuxing***sp. nov.** (♀), *I. taunggyi***sp. nov.** (♂♀) and *I. zhigangi***sp. nov.** (♂♀). Morphological descriptions and photographic illustrations of the new species are given. All types are preserved in the Institute of Zoology, Chinese Academy of Sciences (IZCAS).

## Introduction

The genus *Ischnothyreus* was established by Simon in 1893, with *Ischnaspis peltifer* Simon, 1892 from Saint Vincent in the Caribbean as the type species (Simon 1893). This genus is an Old World taxon, being represented in the New World by only two species, *I. peltifer* and *I. velox* Jackson, 1908, both of which are assumed to be introduced (Platnick et al. 2012; Brescovit et al. 2019).

The genus *Ischnothyreus* Simon, 1893 can be recognized by the presence of leg spines, the usually small abdominal scutum, the strongly sclerotized male palps, the heavily sclerotized male endites, and the winding genital tube in females (Kranz-Baltensperger 2011). There are currently 107 valid specific names assigned to *Ischnothyreus* (Li 2020; WSC 2020) and even more are waiting to be described (Richard et al. 2016).

In this paper seven new *Ischnothyreus* species collected from Myanmar are described and illustrated. This work presents the first record and description of species *Ischnothyreus* from Myanmar.

## Materials and methods

The specimens were examined in 95% ethanol using a Leica M205C stereomicroscope. Details were studied with an Olympus BX51 compound microscope. Photos were taken with a Canon EOS 750D zoom digital camera (18 megapixels) mounted on an Olympus BX51 compound microscope. Vulvae were cleared in lactic acid. Scanning electron microscope images (SEM) were taken under high vacuum with a Hitachi TM3030 after critical point drying and gold-palladium coating. All measurements were taken using an Olympus BX51 compound microscope and are given in millimeters in the text. The specimens are preserved in the Institute of Zoology, Chinese Academy of Sciences (IZCAS) in Beijing, China (curator: Jun Chen).

The following abbreviations are used in the text and figures: **a** = apodemes; **ALE** = anterior lateral eyes; **ass** = anchor-shaped structure; **bsa** = bell-shaped atrium; **csa** = circular atrium; **csp** = crown-shaped sclerotized process; **hsm** = hook-shaped membrane; **llm** = leaf like membrane; **lpp** = leaf-shaped prolateral projection; **nlm** = needle like membrane; **nsa** = nipple-shaped atrium; **oa** = opening of the atrium; **PLE** = posterior lateral eyes; **PME** = posterior median eyes; **rl** = retrolateral lobe; **sem** = serrated exterior margin; **tsa** = triangular shaped atrium; **vpr** = ventral protuberance; **wt** = winding tube.

## Taxonomy

**Family Oonopidae Simon, 1890**

### Genus *Ischnothyreus* Simon, 1893

#### Ischnothyreus hponkanrazi

Taxon classificationAnimaliaAraneaeOonopidae

Tong & Li sp. nov.

DA82F63F-5CAC-5784-A2CC-8447DA05E829

http://zoobank.org/EFA6509C-6C65-46CC-B65D-E54770EAB9E5

Figures 1 , 16A, B

##### Type material.

***Holotype*** ♀: Myanmar, Kachin State, Putao, Hponkanrazi Wildlife Sanctuary, roadside between Camp 2 to Camp 1; 27°36'067"N, 96°59'367"E; elevation ca 1714 m; 17.XII.2016; Wu J. leg. (IZCAS AR-25158).

##### Diagnosis.

**Figure 1.**
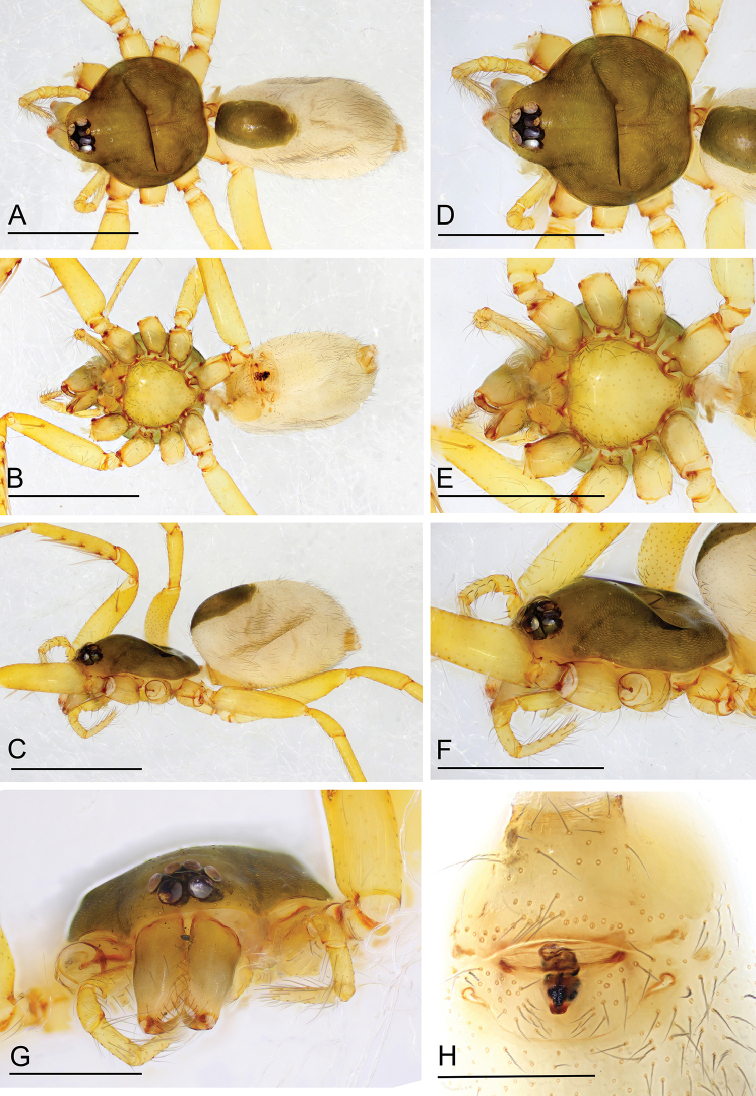
*Ischnothyreus hponkanrazi* sp. nov., female holotype **A–C** habitus, dorsal, ventral and lateral views **D–G** prosoma, dorsal, ventral, lateral and anterior views **H** epigastric region, ventral view. Scale bars: 0.4 mm (**A–F**); 0.2 mm (**G, H**).

The new species is similar to *I. campanaceus* Tong & Li, 2008 in the bell-shaped atrium, but can be distinguished by the short abdominal dorsal scutum (1/3 of the abdomen length (Fig. 1A) vs 4/5 of the abdomen length (Tong and Li 2008: fig. 1B; Tong 2013: fig. 44B), and the greater sinuosity of the winding tube of endogyne (Fig. 1H) (vs short, simple winding tube; Tong and Li 2008: fig. 1F; Tong 2013: fig. 44F).

##### Description.

**Female (holotype). *Body***: habitus as in Fig. 1A–C; body length 2.40. ***Carapace***: 1.08 long, 0.98 wide; pale brown, without any pattern, ovoid in dorsal view, slightly elevated in lateral view, surface finely reticulate, fovea absent, lateral margin straight, smooth (Fig. 1D, F). ***Clypeus***: height about equal to ALE radius or more (Fig. 1G). ***Eyes***: six, in one group, well developed, subequal, ALE circular, PME and PLE oval, posterior eye row procurved from both above and front (Fig. 1D, G). ***Sternum***: as long as wide, pale orange, uniform, not fused to carapace, surface smooth, setae sparse (Fig. 1E). ***Mouthparts***: chelicerae, endites and labium orange; chelicerae straight, base of fangs unmodified (Fig. 1G); labium rectangular, not fused to sternum, anterior margin not indented at middle; endites unmodified (Fig. 1E). ***Abdomen***: 1.33 long, 0.74 wide; dorsal scutum well sclerotized, pale brown, covering 1/3 of the abdomen width and approximately 1/3 of the abdomen length, not fused to epigastric scutum; epigastric and postgastric scutum well sclerotized, pale orange, unfused (Fig. 1A, B). ***Legs***: pale orange, femur I with two prolateral spines, tibia I with four pairs, metatarsus I with two pairs of long ventral spines. Leg II spination is similar to leg I except femur with only one prolateral spine. Legs III and IV spineless. ***Epigastric area***: surface without external features (Fig. 1H). ***Endogyne***: from the middle of the slightly thickened margin of the postgastric scutum runs a dark winding tube posteriorly, ending in a bell-shaped atrium (Fig. 16A, B).

**Male.** Unknown.

##### Etymology.

The specific name is a noun in apposition taken from the type locality.

##### Distribution.

Known only from the type locality.

#### Ischnothyreus jianglangi

Taxon classificationAnimaliaAraneaeOonopidae

Tong & Li sp. nov.

8DF2A223-AE7D-5D8F-95BB-7DE2D6DC9F2B

http://zoobank.org/505F6C65-291C-47C0-8997-23CA63EA5161

Figures 2 , 17A, B

##### Type material.

***Holotype*** ♀: Myanmar, Kachin State, Putao, Hponkanrazi Wildlife Sanctuary, roadside between Camp 2 to Camp 1; 27°35'806"N, 96°59'532"E; elevation ca 1613 m; 10.V.2017; Wu J. & Chen Z. leg. (IZCAS AR-25159).

##### Diagnosis.

**Figure 2.**
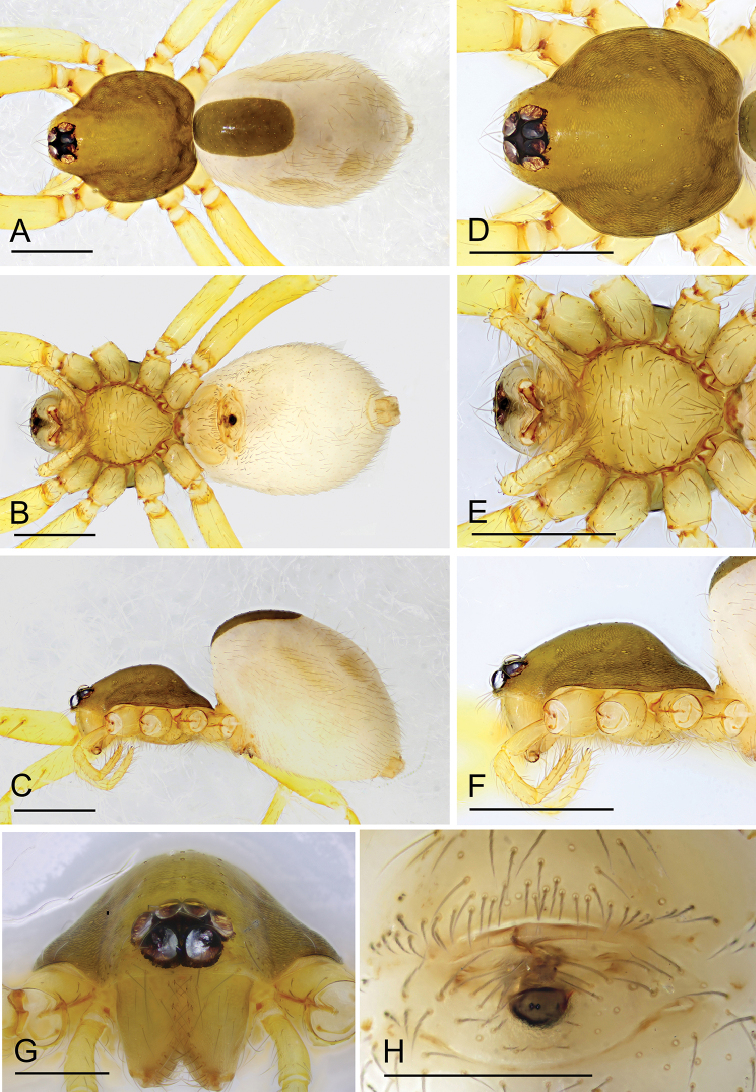
*Ischnothyreus jianglangi* sp. nov., female holotype **A–C** habitus, dorsal, ventral and lateral views **D–G** prosoma, dorsal, ventral, lateral and anterior views **H** epigastric region, ventral view. Scale bars: 0.4 mm (**A–F**); 0.1 mm (**G, H**).

The new species is similar to *I. jojo* Kranz-Baltensperger, 2011 in the triangular shaped atrium, but can be distinguished by shape of the dorsal abdominal scutum (width/length = 1/2 (Fig. 2A) vs approximately 1/3; Kranz-Baltensperger 2011: fig. 25A), and the unmodified exterior surface of postgastric scutum (Fig. 17A) (vs with curved, sclerotized extensions and U-shaped structure; Kranz-Baltensperger 2011: fig. 25E, F).

##### Description.

**Female (holotype). *Body***: habitus as in Fig. 2A–C; body length 2.35. ***Carapace***: 1.02 long, 0.87 wide; brown, without any pattern, ovoid in dorsal view, slightly elevated in lateral view, surface finely reticulate, fovea absent, lateral margin straight, smooth (Fig. 2D, F). ***Clypeus***: height about equal to ALE radius or less (Fig. 2G). ***Eyes***: six, in one group, well developed, subequal, ALE circular, PME and PLE oval, posterior eye row procurved from both above and front (Fig. 2D, G). ***Sternum***: as long as wide, pale brown, uniform, not fused to carapace, surface smooth, setae sparse (Fig. 2E). ***Mouthparts***: chelicerae, endites and labium pale brown; chelicerae straight, base of fangs unmodified (Fig. 2G); labium rectangular, not fused to sternum, anterior margin not indented at middle; endites unmodified (Fig. 2E). ***Abdomen***: 1.42 long, 1.01 wide; dorsal scutum covering less than 1/2 of the abdomen length and 1/3 of the abdomen width, not fused to epigastric scutum; epigastric and postgastric scutum well sclerotized, pale orange, unfused (Fig. 2A, B). **Legs**: pale orange, femur I with two prolateral spines, tibia I with four pairs, metatarsus I with two pairs of long ventral spines. Leg II spination is similar to leg I except femur with only one prolateral spine. Legs III and IV spineless. ***Epigastric area***: surface without external features (Fig. 2H). ***Endogyne***: from the middle of the slightly thickened margin of the postgastric scutum runs a dark, simple winding tube posteriorly, ending in a black, triangular shaped atrium (Fig. 17A, B).

**Male.** Unknown.

##### Etymology.

The species is named after Mr Jianglang Wu, one of the collectors of the holotype; noun in genitive case.

##### Distribution.

Known only from the type locality.

#### Ischnothyreus meukyawwa

Taxon classificationAnimaliaAraneaeOonopidae

Tong & Li sp. nov.

C875D286-3D19-56E3-8E0F-33DA051F819B

http://zoobank.org/08A2A68D-453F-4E7F-8F52-05FB0C49EDAC

**Figure 3.**
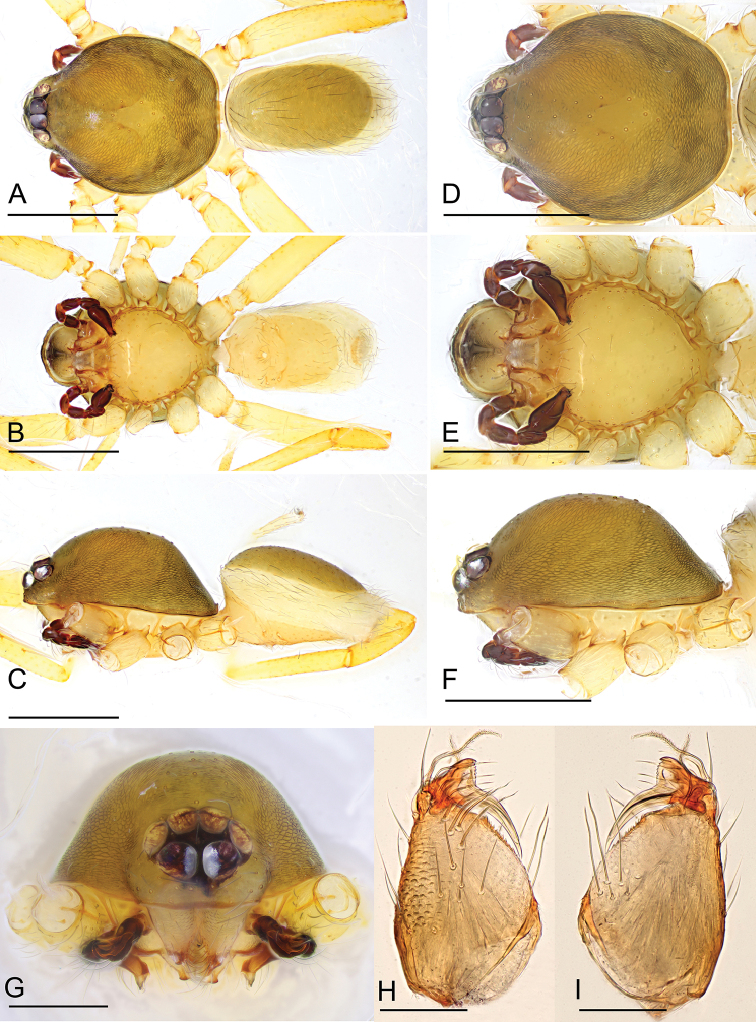
*Ischnothyreus meukyawwa* sp. nov., male holotype **A–C** habitus, dorsal, ventral and lateral views **D–G** prosoma, dorsal, ventral, lateral and anterior views **H, I** left chelicerae, anterior and posterior views. Scale bars: 0.4 mm (**A–F**); 0.2 mm (**G**); 0.1 mm (**H, I**).

**Figure 4.**
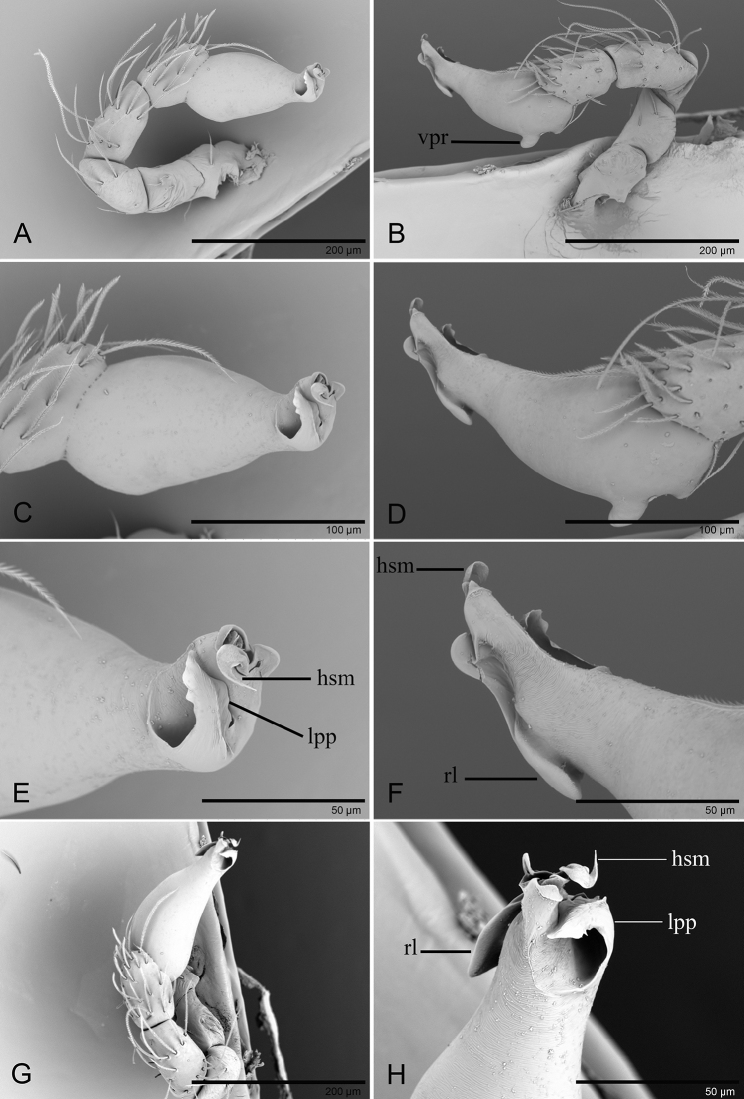
*Ischnothyreus meukyawwa* sp. nov., male holotype, left palp, SEM**A, B, G** prolateral, retrolateral and dorsal views **C, D** palpal bulb, prolateral and retrolateral views **E, F, H** distal part of palpal bulb, prolateral, retrolateral and dorsal views. Abbreviations: hsm = hook-shaped membrane; lpp = leaf-shaped prolateral projection; rl = retrolateral lobe; vpr = ventral protuberance.

**Figure 5.**
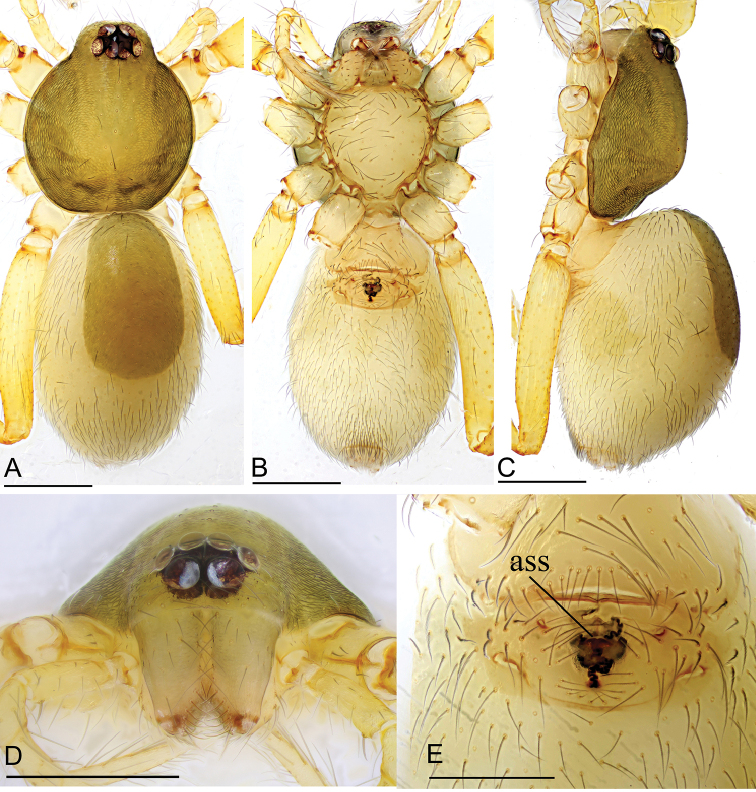
*Ischnothyreus meukyawwa* sp. nov., female paratype **A–C** habitus, dorsal, ventral and lateral views **D** prosoma, anterior view **E** epigastric region, ventral view. Abbreviation: **ass** = anchor-shaped structure. Scale bars: 0.4 mm (**A–C**); 0.2 mm (**D, E**).

Figures 3 , 4 , 5 , 14A–C , 15A, B , 16C, D

##### Type material.

***Holotype*** ♂: Myanmar, Kachin State, Putao, Meukyawwa Village; 27°20'883"N, 97°22'717"E; elevation ca 464 m; 25.XII.2016; Wu J. leg. (IZCAS AR-25160). ***Paratypes***: 5♂ 9♀, same data as for holotype (IZCAS AR-25161–25174).

##### Diagnosis.

The new species is similar to *I. an* Tong & Li, 2016 in the large abdominal dorsal and ventral scutum, but can be distinguished by the unmodified male cheliceral fang (Fig. 3H, I) (vs with thorn-like protrusion; Tong et al. 2016: fig. 1G, H), the acute distal end of male palp (Fig. 14A) (vs blunt distal end of male palp; Tong et al. 2016: fig. 3A, D), and the small bell-shaped atrium (Fig. 16C) (vs a large equilateral triangular shaped atrium; Tong et al. 2016: fig. 2G, I).

##### Description.

**Male (holotype). *Body***: habitus as in Fig. 3A–C; body length 1.57. ***Carapace***: 0.89 long, 0.74 wide; pale brown, with egg-shaped patches behind eyes, ovoid in dorsal view, strongly elevated in lateral view, surface of elevated portion of pars cephalica smooth, sides finely reticulate, fovea absent, lateral margin straight, smooth (Fig. 3D, F). ***Clypeus***: height about 2/3 of ALE diameter (Fig. 3G). ***Eyes***: six, in one group, well developed, subequal, ALE circular, PME and PLE oval, posterior eye row recurved from above, procurved from front (Fig. 3D, G). ***Sternum***: as long as wide, pale orange, uniform, not fused to carapace, surface smooth, setae sparse (Fig. 3E). ***Mouthparts***: chelicerae, endites and labium orange; chelicerae straight, base of fangs with crown-shaped sclerotized process with serrated exterior margin, fang groove with a few small denticles (Figs 3H, I, 15A, B); labium rectangular, not fused to sternum, anterior margin not indented at middle; anteromedian tip of endites with one strong, tooth-like projection (Fig. 3E). ***Abdomen***: 0.84 long, 0.43 wide; dorsal scutum well sclerotized, pale orange, covering whole abdomen width and approximately 5/6 of the abdomen length, not fused to epigastric scutum; epigastric and postgastric scutum well sclerotized, pale orange, fused, postgastric scutum covering about 5/6 of the abdomen length (Fig. 3A–C). ***Legs***: pale orange, femur I with three prolateral and two small retrolateral spines, tibia I with four pairs, metatarsus I with two pairs of long ventral spines. Leg II spination is similar to leg I except femur with only two prolateral and one retrolateral spine. Legs III and IV spineless. ***Palp***: strongly sclerotized, trochanter with ventral projection, cymbium brown, fused with bulb; bulb brown, with one large and one very small ventral protuberance, distal end of bulb elongated, with one broad leaf-shaped prolateral projection and distal hook-shaped membrane, retrolateral lobe narrow (Figs 4, 14A–C).

**Female (paratype, IZCAS AR-25160).** Same as male except as noted. ***Body***: habitus as in Fig. 5A–C; body length 1.93. **Carapace**: 0.89 long, 0.76 wide; without any pattern. ***Mouthparts***: chelicerae and endites unmodified. ***Abdomen***: 0.83 long, 0.75 wide; dorsal scutum covering 3/5 of the abdomen length, about 1/2 of the abdomen width. ***Epigastric area***: the postgastric scutum with central anchor-shaped structure (Fig. 5E). ***Endogyne***: from the middle of the slightly thickened margin of the postgastric scutum runs a dark, simple winding tube posteriorly, ending in a small bell-shaped atrium (Fig. 16C, D).

##### Etymology.

The specific name is a noun in apposition taken from the type locality.

##### Distribution.

Known only from the type locality.

#### Ischnothyreus putao

Taxon classificationAnimaliaAraneaeOonopidae

Tong & Li sp. nov.

22E363FD-2894-5B9E-A8D7-752E16ED9A5D

http://zoobank.org/D96DFBF0-A942-44A3-833A-18EB8FB325B1

Figures 6 , 17C, D

##### Type material.

***Holotype*** ♀: Myanmar, Kachin State, Putao, Hponkanrazi Wildlife Sanctuary, roadside between Camp 1 to Camp 2; 27°36'567"N, 96°.58'850"E; elevation ca 2233 m; 15.XII.2016; Wu J. leg. (IZCAS AR-25175).

##### Diagnosis.

**Figure 6.**
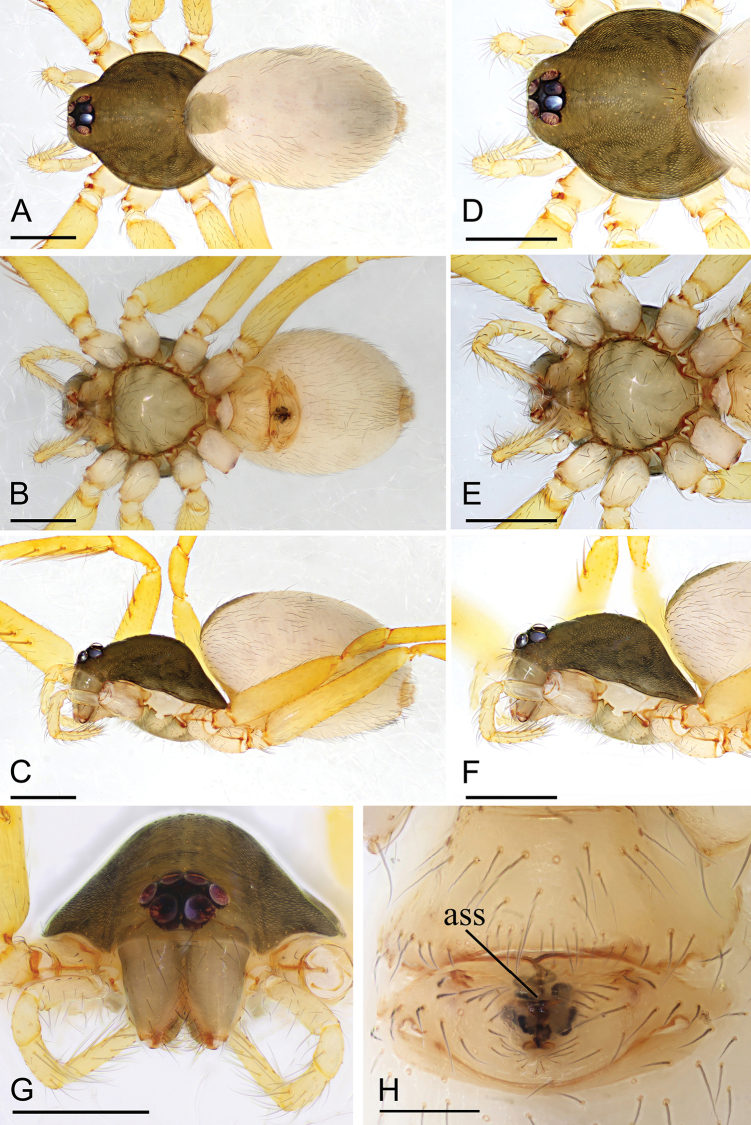
*Ischnothyreus putao* sp. nov., female holotype **A–C** habitus, dorsal, ventral and lateral views **D–G** prosoma, dorsal, ventral, lateral and anterior views **H** epigastric region, ventral view. Abbreviation: ass = anchor-shaped structure. Scale bars: 0.4 mm (**A–F**); 0.2 mm (**G**); 0.1 mm (**H**).

The new species is similar to *I. zhigangi* sp. nov. in the very small abdominal dorsal scutum and the brown carapace, but can be distinguished by the large bell-shaped atrium (Fig. 17C) (vs small, bell-shaped atrium; Fig. 17E), and the smoothly curved posterior margin of postgastric scutum (Fig. 6H) (vs straight posterior margin; Fig. 13H).

##### Description.

**Female (holotype). *Body***: habitus as in Fig. 6A–C; body length 1.94. ***Carapace***: 0.97 long, 0.88 wide; dark brown, without any pattern, ovoid in dorsal view, strongly elevated in lateral view, surface finely reticulate, fovea absent, lateral margin straight, smooth (Fig. 6D, F). ***Clypeus***: height about equal to ALE radius or more (Fig. 6G). ***Eyes***: six, in one group, well developed, subequal, ALE circular, PME and PLE oval, posterior eye row procurved from both above and front (Fig. 6D, G). ***Sternum***: as long as wide, pale brown, uniform, not fused to carapace, surface smooth, setae sparse (Fig. 6E). ***Mouthparts***: chelicerae, endites and labium brown; chelicerae straight, base of fangs unmodified (Fig. 6G); labium rectangular, not fused to sternum, anterior margin not indented at middle; endites unmodified (Fig. 6E). ***Abdomen***: 1.30 long, 0.89 wide; dorsal scutum weakly sclerotized, very small, not fused to epigastric scutum; epigastric and postgastric scutum well sclerotized, pale orange, unfused (Fig. 6A, B). ***Legs***: pale orange, femur I with three prolateral spines, tibia I with four pairs, metatarsus I with two pairs of long ventral spines. Leg II spination is similar to leg I except femur with only two prolateral spine. Legs III and IV spineless. ***Epigastric area***: the postgastric scutum with central, anchor-shaped structure, and smoothly curved posterior margin (Fig. 6H). ***Endogyne***: from the middle of the slightly thickened margin of the postgastric scutum runs a dark, simple winding tube posteriorly, ending in a large, inverted bell-shaped atrium (Fig. 17C, D).

**Male.** Unknown.

##### Etymology.

The specific name is a noun in apposition taken from the type locality.

##### Distribution.

Known only from the type locality.

#### Ischnothyreus qiuxing

Taxon classificationAnimaliaAraneaeOonopidae

Tong & Li sp. nov.

F05998A5-3D55-51D3-A043-C5573DA67D9B

http://zoobank.org/8B8870A1-6AD1-4C30-B8E3-616837C148B7

Figures 7 , 16E, F

##### Type material.

***Holotype*** ♀: Myanmar, Kachin State, Putao, Around Ziradum Village; 27°33'465"N, 97°06'580"E; elevation ca 1051 m; 8.V.2017; Wu J. & Chen Z. leg. (IZCAS AR-25176).

##### Diagnosis.

The new species is similar to *I. balu* Kranz-Baltensperger, 2011 in the circular atrium, but can be distinguished by the size of atrium (nearly 1/5 the length of postgastric scutum (Fig. 16E) vs more than 1/3 the length of postgastric scutum; Kranz-Baltensperger 2011: fig. 2F, H) and the greater sinuosity of the winding tube (Fig. 16F) (vs short, simple winding tube; Kranz-Baltensperger 2011: fig. 2G).

##### Description.

**Figure 7.**
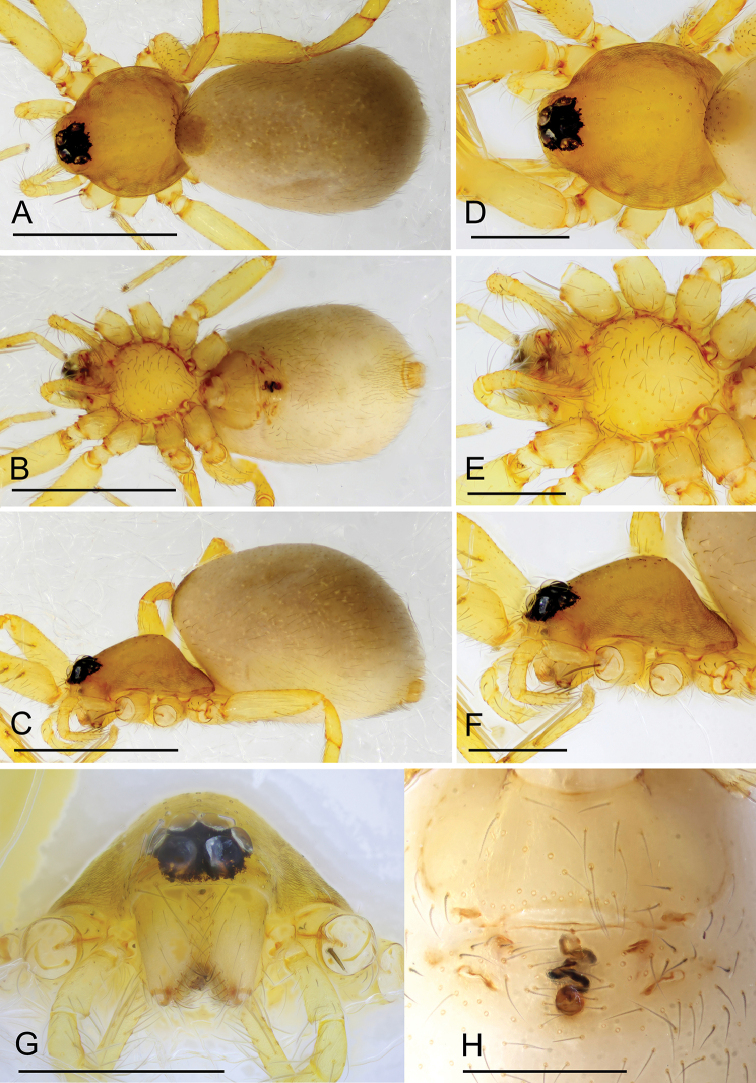
*Ischnothyreus qiuxing* sp. nov., female holotype **A–C** habitus, dorsal, ventral and lateral views **D–G** prosoma, dorsal, ventral, lateral and anterior views **H** epigastric region, ventral view. Scale bars: 0.4 mm (**A–C**); 0.2 mm (**D–H**).

**Female (holotype). *Body***: habitus as in Fig. 7A–C; body length 2.01. ***Carapace***: 0.87 long, 0.72 wide; yellow, without any pattern, ovoid in dorsal view, strongly elevated in lateral view, surface finely reticulate, fovea absent, lateral margin straight, smooth (Fig. 7D, F). ***Clypeus***: height about equal to ALE radius or less (Fig. 7G). ***Eyes***: six, in one group, well developed, subequal, ALE circular, PME and PLE oval, posterior eye row recurved from above, procurved from front (Fig. 7D, G). ***Sternum***: as long as wide, yellow, uniform, not fused to carapace, surface smooth, setae sparse (Fig. 7E). ***Mouthparts***: chelicerae, endites and labium orange; chelicerae straight, base of fangs unmodified (Fig. 7G); labium rectangular, not fused to sternum, anterior margin not indented at middle; endites unmodified (Fig. 7E). ***Abdomen***: 1.35 long, 0.92 wide; dorsal scutum weakly sclerotized, very small, not fused to epigastric scutum; epigastric and postgastric scutum well sclerotized, pale orange, unfused (Fig. 7A, B). ***Legs***: pale orange, femur I with two prolateral spines, tibia I with four pairs, metatarsus I with two pairs of long ventral spines. Leg II spination is similar to leg I except femur with only one prolateral spine. Legs III and IV spineless. ***Epigastric area***: surface without external features (Fig. 7H). ***Endogyne***: from the middle of the slightly thickened margin of the postgastric scutum runs a dark, very simple winding tube posteriorly, ending in a circular atrium (Fig. 16E, F).

**Male.** Unknown.

##### Etymology.

The specific name is derived from Chinese pinyin, “qiuxing”, which means “circular”, referring to the circular atrium; noun in apposition.

##### Distribution.

Known only from the type locality.

#### Ischnothyreus taunggyi

Taxon classificationAnimaliaAraneaeOonopidae

Tong & Li sp. nov.

F02A79E6-777F-5435-B523-FDF6866D1E14

http://zoobank.org/E8228172-15A8-4BCB-8B68-BC87903BCBDE

**Figure 8.**
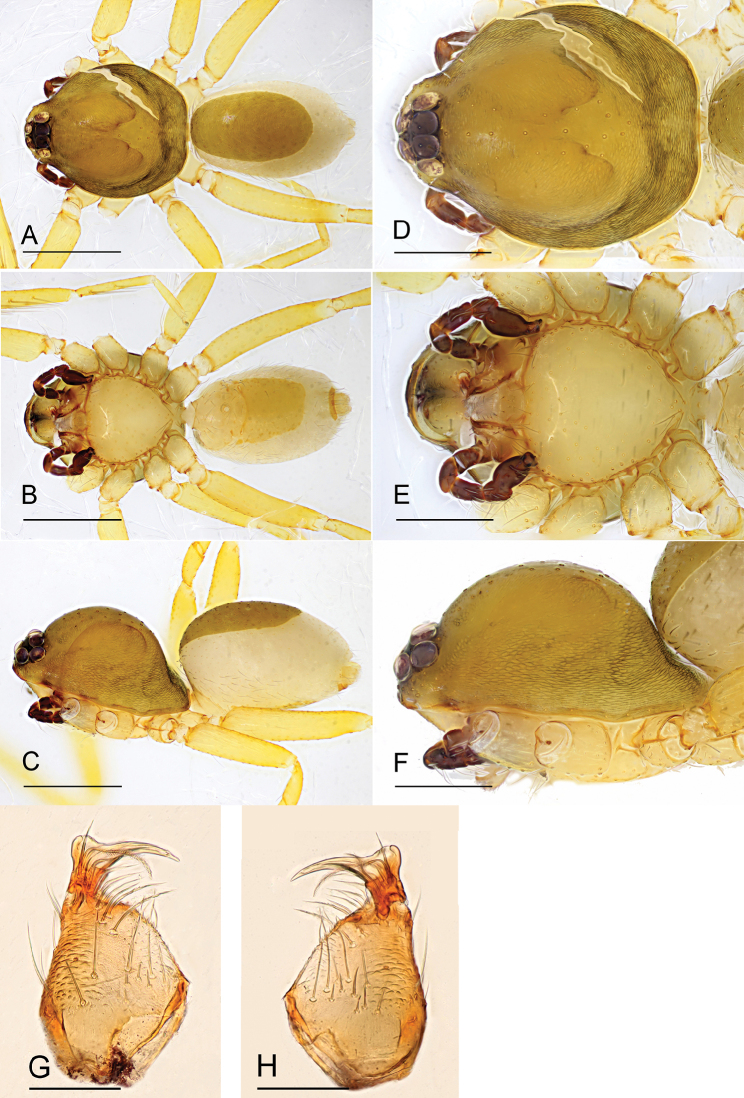
*Ischnothyreus taunggyi* sp. nov., male holotype **A–C** habitus, dorsal, ventral and lateral views **D–F** prosoma, dorsal, ventral and lateral views **G, H** left chelicerae, anterior and posterior views. Scale bars: 0.4 mm (**A–C**); 0.2 mm (**D–F**); 0.1 mm (**G, H**).

**Figure 9.**
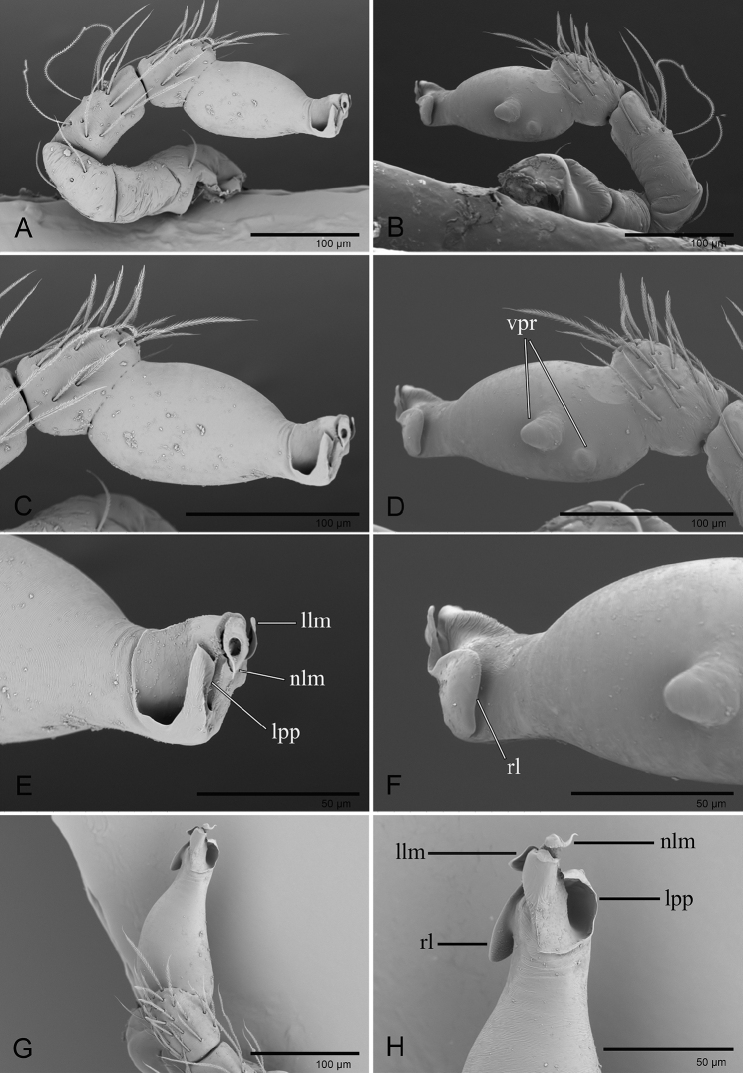
*Ischnothyreus taunggyi* sp. nov., male holotype, left palp, SEM**A, B** prolateral and retrolateral views **C, D, G** palpal bulb, prolateral, retrolateral and dorsal views **E, F, H** distal part of palpal bulb, prolateral, retrolateral and dorsal views. Abbreviations: nlm = needle like membrane; llm = leaf like membrane; lpp = leaf-shaped prolateral projection; rl = retrolateral lobe; vpr = ventral protuberance.

**Figure 10.**
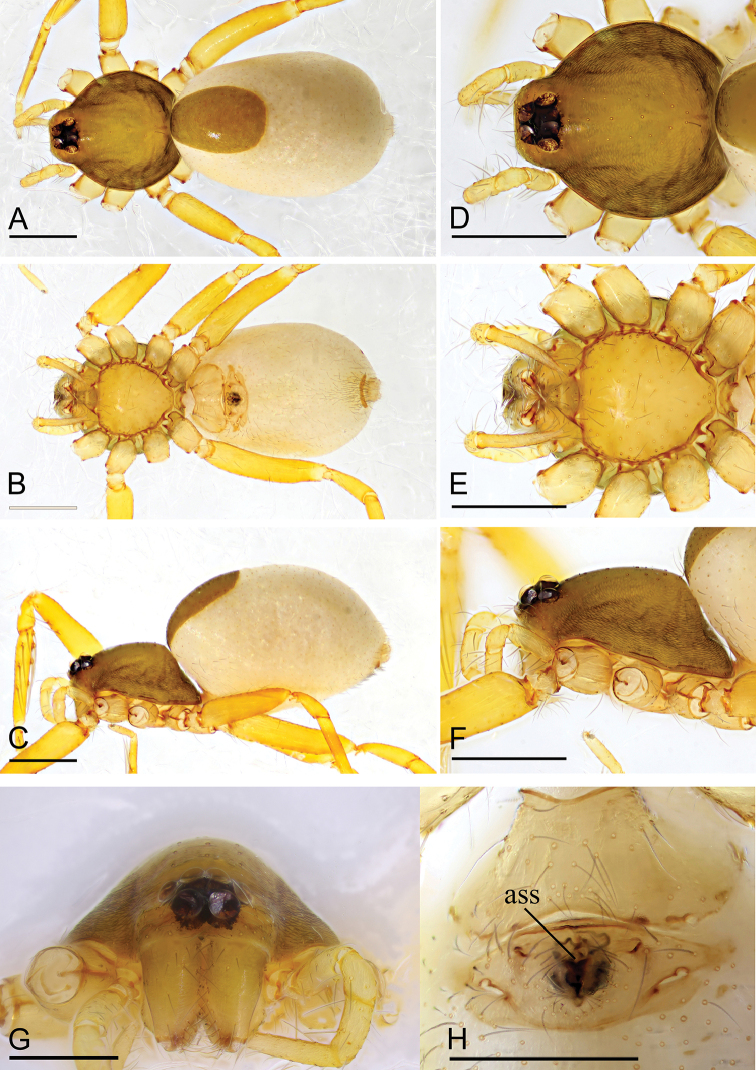
*Ischnothyreus taunggyi* sp. nov., female paratype **A–C** habitus, dorsal, ventral and lateral views **D–F, G** prosoma, dorsal, ventral, lateral and anterior views **H** epigastric region, ventral view. Abbreviation: ass = anchor-shaped structure. Scale bars: 0.4 mm (**A–F**); 0.2 mm (**G, H**).

Figures 8 , 9 , 10 , 14D–F , 15C, D , 16G, H

##### Type material.

***Holotype*** ♂: Myanmar, Shan State, Taunggyi, East of Nyaung Shwe Township; 20°34'700"N, 96°57'450"E; elevation ca 1005 m; 30.XI.2016; Wu J. leg. (IZCAS AR-25177). ***Paratypes*** 2♀: same data as for holotype (IZCAS AR-25178–25179).

##### Diagnosis.

**Figure 11.**
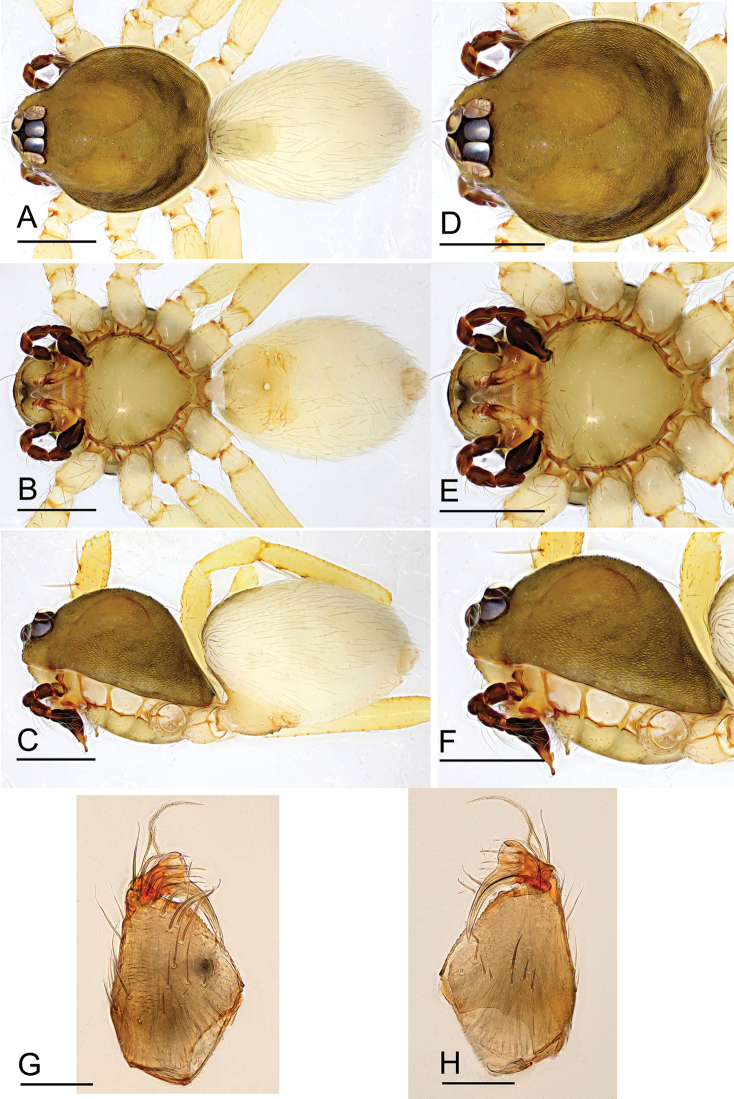
*Ischnothyreus zhigangi* sp. nov., male holotype **A–C** habitus, dorsal, ventral and lateral views **D–F** prosoma, dorsal, ventral and lateral views **G, H** left chelicerae, anterior and posterior views. Scale bars: 0.4 mm (**A–F**); 0.1 mm (**G, H**).

The new species is similar to *I. zhigangi* sp. nov. in the male palp and the crown-shaped sclerotized process of male cheliceral fang, but can be distinguished by the long abdominal dorsal scutum (3/4 of the abdomen length (Fig. 8A) vs very small; Fig. 11A) and ventral scutum (4/5 of the abdomen length (Fig. 8B) vs very small; Fig. 11B) of male, and the long abdominal dorsal scutum (less than 1/2 of the abdomen length (Fig. 10A) vs very small; Fig. 13A) and the nipple-shaped atrium (Fig. 16G) (vs inverted bell-shaped atrium; Fig. 17E) of female.

##### Description.

**Male (holotype). *Body***: habitus as in Fig. 8A–C; body length 1.71. ***Carapace***: 0.84 long, 0.63 wide; pale brown, with egg-shaped patches behind eyes, ovoid in dorsal view, strongly elevated in lateral view, surface of elevated portion of pars cephalica smooth, sides finely reticulate, fovea absent, lateral margin straight, smooth (Fig. 8D). ***Clypeus***: height about equal to ALE radius or more. ***Eyes***: six, in one group, well developed, ALE largest, ALE circular, PME and PLE oval, posterior eye row recurved from above, procurved from front (Fig. 8D). ***Sternum***: as long as wide, pale orange, uniform, not fused to carapace, surface smooth, setae sparse (Fig. 8E). ***Mouthparts***: chelicerae, endites and labium orange; chelicerae straight, with crown-shaped sclerotized process at base of fangs, fang groove with a few small and one larger denticles (Fig. 15C, D); labium rectangular, not fused to sternum, anterior margin not indented at middle; anteromedian tip of endites with one strong, tooth-like projection (Fig. 8E). ***Abdomen***: 0.74 long, 0.49 wide; dorsal scutum well sclerotized, pale orange, covering 2/3 the abdomen width and approximately 3/4 of the abdomen length, unfused to epigastric scutum; epigastric and postgastric scutum well sclerotized, pale orange, fused, postgastric scutum covering about 4/5 of the abdomen length (Fig. 8A, B). ***Legs***: pale orange, femur I with three prolateral and one small retrolateral spines, tibia I with four pairs, metatarsus I with two pairs of long ventral spines. Leg II spination is similar to leg I except femur with only two prolateral spines. Legs III and IV spineless. ***Palp***: strongly sclerotized, trochanter with ventral projection, cymbium brown, fused with bulb; bulb brown, with two large ventral protuberances, distal end of bulb elongated, with one leaf-shaped prolateral projection and distal needle like membrane, retrolateral lobe broad, ear-shaped (Figs 9, 14D–F).

**Female (paratype, IZCAS AR-25178).** Same as male except as noted. ***Body***: habitus as in Fig. 10A–C; body length 1.98. **Carapace**: 0.83 long, 0.71 wide; without any pattern (Fig. 10D). ***Mouthparts***: chelicerae and endites unmodified (Fig. 10E, G). ***Abdomen***: 1.27 long, 0.83 wide; dorsal scutum covering less than 1/2 of the abdomen length, about 1/3 of the abdomen width (Fig. 10A). ***Epigastric area***: the postgastric scutum with central anchor-shaped structure (Fig. 10H). ***Endogyne***: from the middle of the slightly thickened margin of the postgastric scutum runs a dark, very complex winding tube posteriorly, ending in a small, bell-shaped atrium (Fig. 16G, H).

##### Etymology.

The specific name is a noun in apposition taken from the type locality.

##### Distribution.

Known only from the type locality.

#### Ischnothyreus zhigangi

Taxon classificationAnimaliaAraneaeOonopidae

Tong & Li sp. nov.

D1101392-585B-5971-8AA8-30EE9DDFBF19

http://zoobank.org/CB6C29F2-AC7C-4511-8F20-7771D82CB329

**Figure 12.**
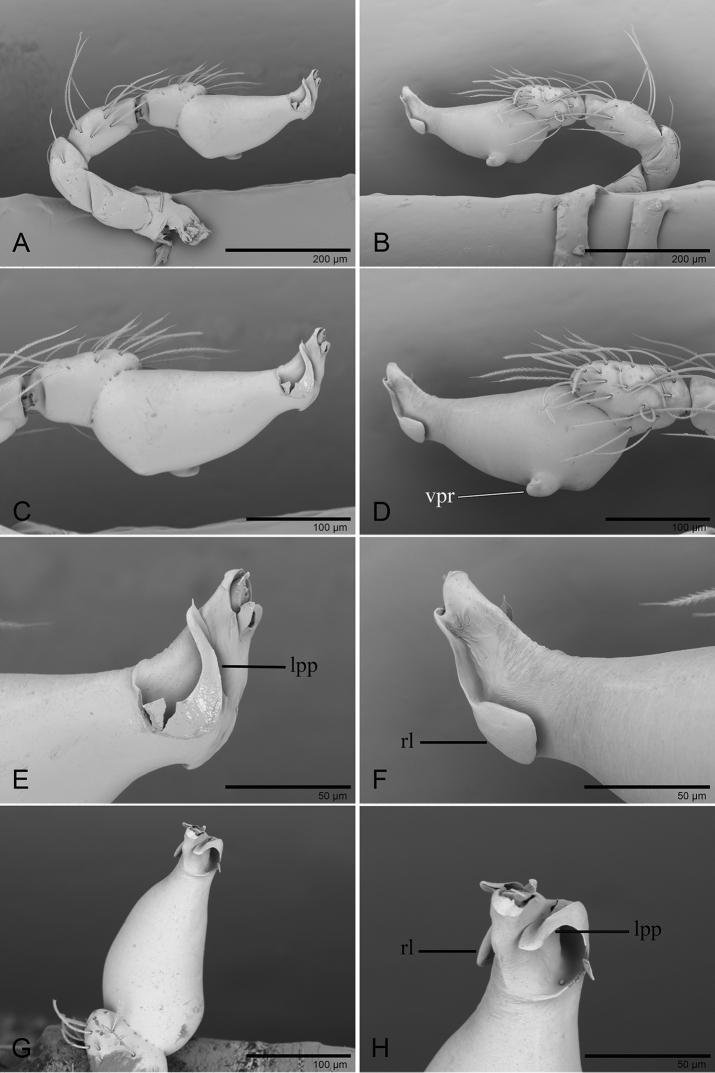
*Ischnothyreus zhigangi* sp. nov., male holotype, left palp, SEM**A, B** prolateral and retrolateral views **C, D, G** palpal bulb, prolateral, retrolateral and dorsal views **E, F, H** distal part of palpal bulb, prolateral, retrolateral and dorsal views. Abbreviations: lpp = leaf-shaped prolateral projection; rl = retrolateral lobe; vpr = ventral protuberance.

**Figure 13.**
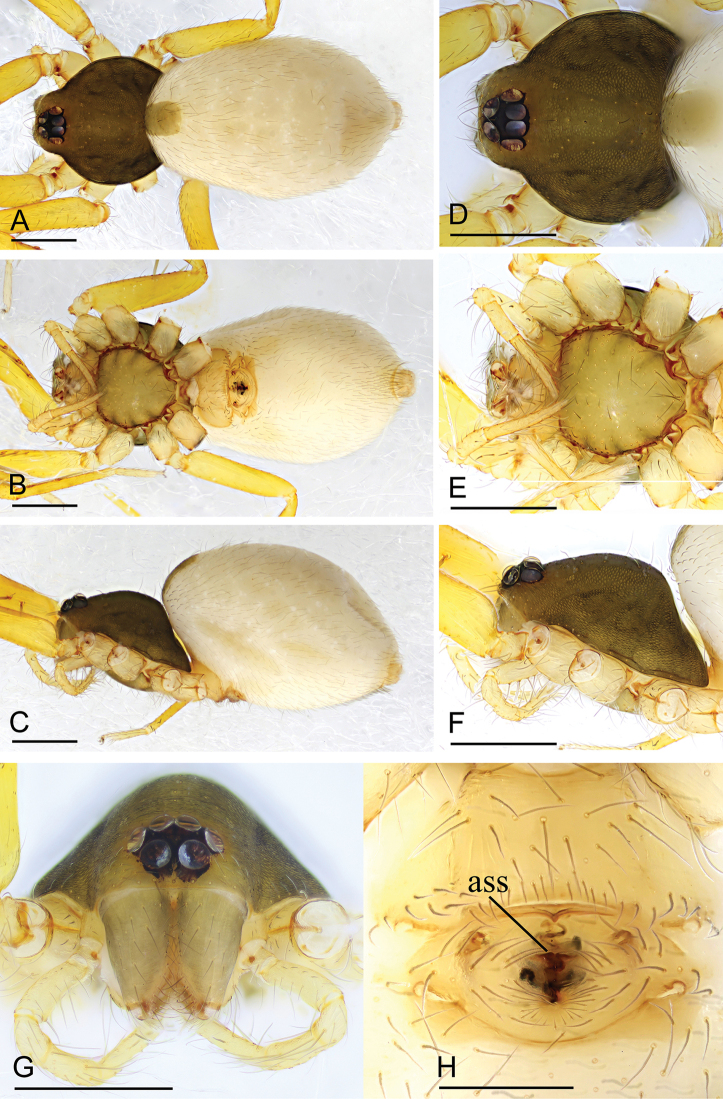
*Ischnothyreus zhigangi* sp. nov., female paratype **A–C** habitus, dorsal, ventral and lateral views **D–G** prosoma, dorsal, ventral, lateral and anterior views **H** epigastric region, ventral view. Abbreviation: ass = anchor-shaped structure. Scale bars: 0.4 mm (**A–G**); 0.1 mm (**H**).

**Figure 14.**
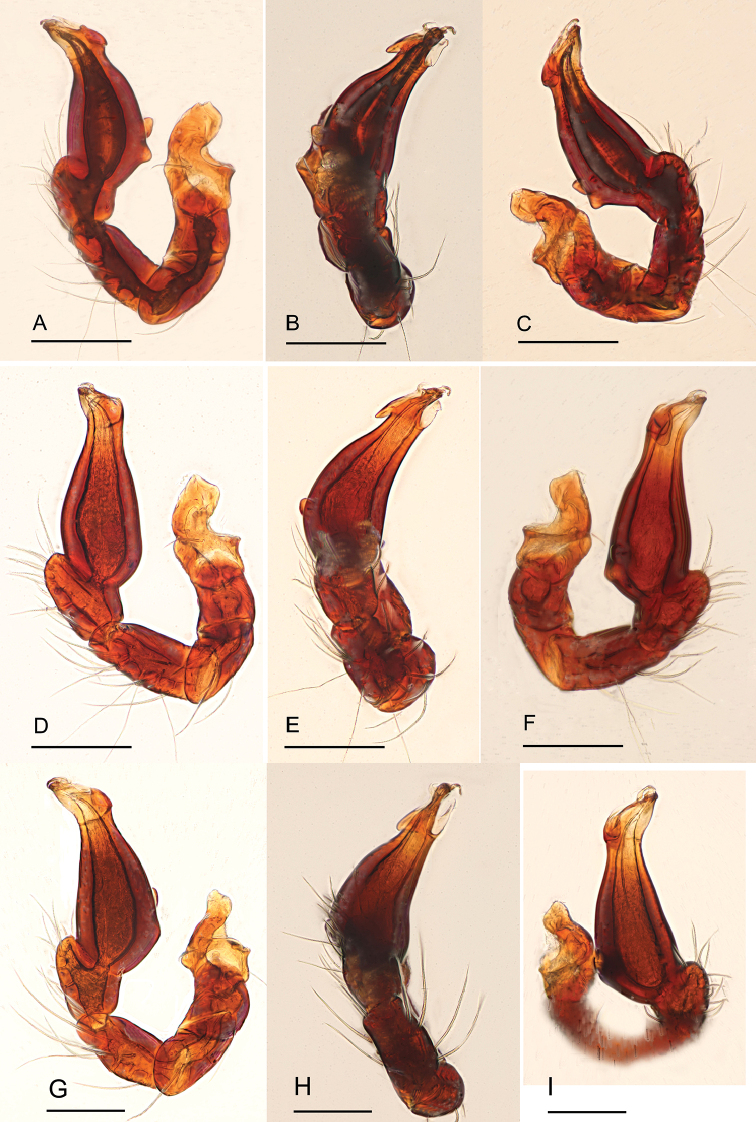
*Ischnothyreus* spp., left male palp **A–C***I. meukyawwa* sp. nov. **D–F***I. taunggyi* sp. nov. **G–I***I. zhigangi* sp. nov. **A, D, G** prolateral view **B, E, H** dorsal view **C, F, I** retrolateral view. Scale bars: 0.1 mm.

**Figure 15.**
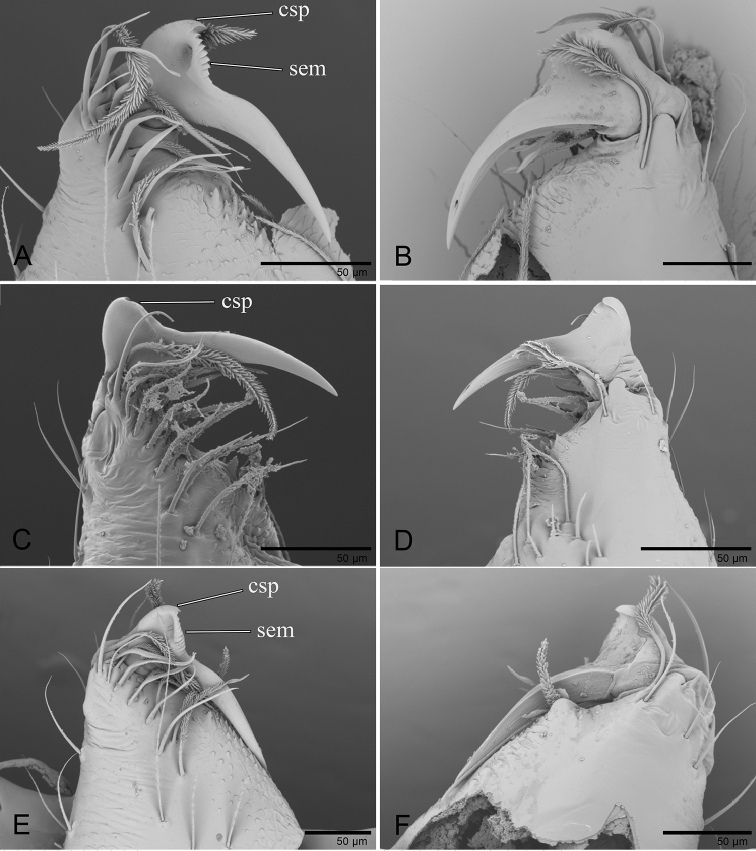
*Ischnothyreus* spp., left male chelicerae. **A, B***I. meukyawwa* sp. nov. **C, D***I. taunggyi* sp. nov. **E, F***I. zhigangi* sp. nov. **A, C, E** anterior view **B, D, F** posterior view. Abbreviations: csp = crown-shaped sclerotized process; sem = serrated exterior margin.

Figures 11 , 12 , 13 , 14G–I , 15E, F , 17E, F

##### Type material

**. *Holotype*** ♂: Myanmar, Putao, Hponkanrazi Wildlife Sanctuary Around Camp 2; 27°36'681"N, 96°58'958"E; elevation ca 2457 m; 11.V.2017; Wu J. & Chen Z. leg. (IZCAS AR-25178). ***Paratypes*** 4♀: data same as for holotype; 27°31'103"N, 96°57'694"E; elevation ca 2737 m; 16.V.2017; Wu J. & Chen Z. leg. (IZCAS AR-25179–25182).

##### Diagnosis.

**Figure 16.**
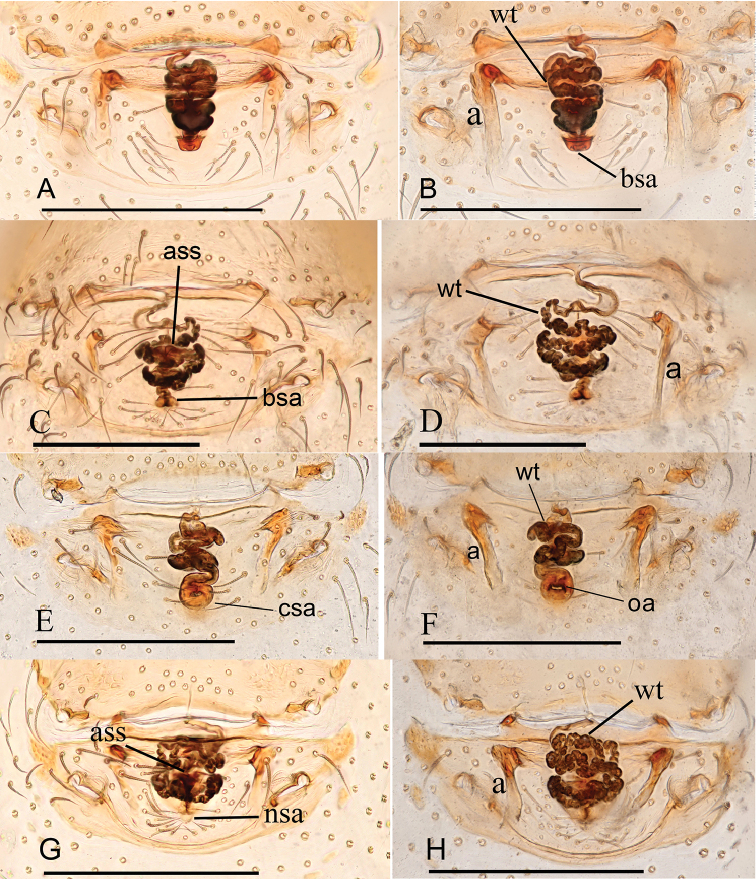
*Ischnothyreus* spp., female copulatory organ **A, B***I. hponkanrazi* sp. nov. **C, D***I. meukyawwa* sp. nov. **E, F***I. qiuxing* sp. nov. **G, H***I. taunggyi* sp. nov. **A, C, E, G** ventral view **B, D, F, H** dorsal view. Abbreviations: a = apodemes; ass = anchor-shaped structure; bsa = bell-shaped atrium; csa = circular atrium; nsa = nipple-shaped atrium; oa = opening of the atrium; wt = winding tube. Scale bars: 0.2 mm.

**Figure 17.**
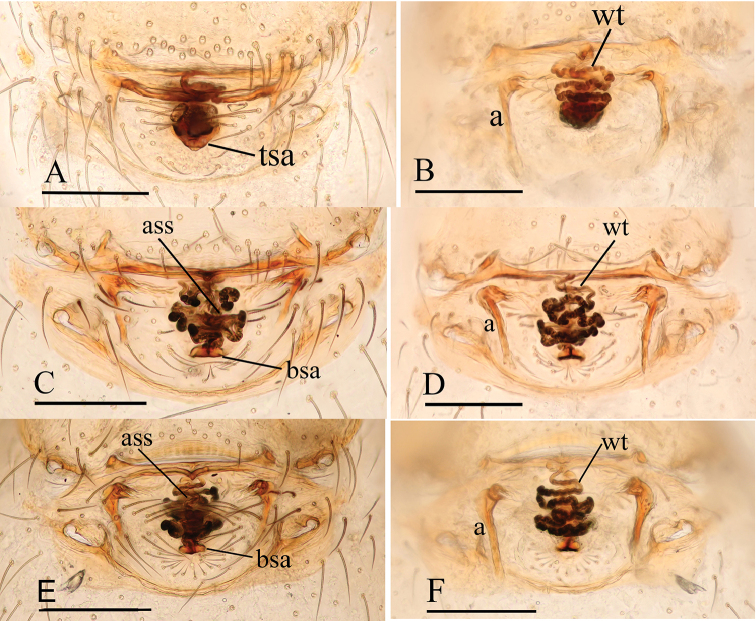
*Ischnothyreus* spp., female copulatory organ **A, B***I. jianglangi* sp. nov. **C, D***I. putao* sp. nov. **E, F***I. zhigangi* sp. nov. **A, C, E** ventral view **B, D, F** dorsal view. Abbreviations: a = apodemes; ass = anchor-shaped structure; bsa = bell-shaped atrium; tsa = triangular shaped atrium; wt = winding tube. Scale barss: 0.1 mm

The new species is similar to *I. taunggyi* sp. nov. but can be distinguished by the short abdominal dorsal scutum (very small (Fig. 11A) vs 3/4 of the abdomen length; Fig. 8A) and ventral scutum (very small (Fig. 11B) vs 4/5 of the abdomen length; Fig. 8B) of male, and the short abdominal dorsal scutum (very small (Fig. 13A) vs less than 1/2 of the abdomen length; Fig. 10A) and the inverted bell-shaped atrium (Fig. 17E) (vs nipple-shaped atrium (Fig. 16G) of female).

##### Description.

**Male (holotype). *Body***: habitus as in Fig. 11A–C; body length 2.08. ***Carapace***: 1.03 long, 0.87 wide; pale brown, with egg-shaped patches behind eyes, ovoid in dorsal view, strongly elevated in lateral view, surface of elevated portion of pars cephalica smooth, sides finely reticulate, fovea absent, lateral margin straight, smooth (Fig. 11D). ***Clypeus***: height about equal to ALE radius or more. ***Eyes***: six, in one group, well developed, subequal, ALE circular, PME and PLE oval, posterior eye row straight from above, procurved from front (Fig. 11D). ***Sternum***: as long as wide, pale brown, uniform, not fused to carapace, surface smooth, setae sparse (Fig. 11E). ***Mouthparts***: chelicerae, endites and labium orange; chelicerae straight, base of fangs with crown-shaped sclerotized process with serrated exterior margin (Figs 11G, H, 15E, F), fang groove with a few small denticles; labium rectangular, not fused to sternum, anterior margin not indented at middle; anteromedian tip of endites with one strong, tooth-like projection (Fig. 11E). ***Abdomen***: 1.07 long, 0.72 wide; dorsal scutum weakly sclerotized, pale orange, very small, not fused to epigastric scutum; epigastric and postgastric scutum weakly sclerotized, pale orange, fused, postgastric scutum very small (Fig. 11A, B). ***Legs***: pale orange, femur I with three prolateral spines, tibia I with four pairs, metatarsus I with two pairs of long ventral spines. Leg II spination is similar to leg I except femur with only two prolateral spines. Legs III and IV spineless. ***Palp***: strongly sclerotized, trochanter with ventral projection, cymbium brown, fused with bulb; bulb brown, with one large ventral protuberance, distal end of bulb elongated, with one narrow leaf-shaped prolateral projection, retrolateral lobe small, simple (Figs 12, 14G–H).

**Female (paratype, IZCAS AR-25179).** Same as male except as noted. ***Body***: habitus as in Fig. 13A–C; body length 2.63. ***Carapace***: 1.10 long, 0.95 wide; without any pattern, posterior eye row procurved from both above and front (Fig. 13D, G). ***Mouthparts***: chelicerae and endites unmodified (Fig. 13E, G). ***Abdomen***: 1.82 long, 1.19 wide. ***Epigastric area***: the postgastric scutum with central anchor-shaped structure (Fig. 13H). ***Endogyne***: from the middle of the slightly thickened margin of the postgastric scutum runs a dark, simple winding tube posteriorly, ending in a small, inverted bell-shaped atrium (Fig. 17E, F).

##### Etymology.

The species is named after Mr Zhigang Chen, one of the collectors of the holotype; noun in genitive case.

##### Distribution.

Known only from the type locality.

## Supplementary Material

XML Treatment for Ischnothyreus hponkanrazi

XML Treatment for Ischnothyreus jianglangi

XML Treatment for Ischnothyreus meukyawwa

XML Treatment for Ischnothyreus putao

XML Treatment for Ischnothyreus qiuxing

XML Treatment for Ischnothyreus taunggyi

XML Treatment for Ischnothyreus zhigangi
